# Cost estimates of HIV care and treatment with and without anti-retroviral therapy at Arba Minch Hospital in southern Ethiopia

**DOI:** 10.1186/1478-7547-7-6

**Published:** 2009-04-13

**Authors:** Asfaw Demissie Bikilla, Degu Jerene, Bjarne Robberstad, Bernt Lindtjorn

**Affiliations:** 1 Center for International Health, University of Bergen, PO Box 7804, 5020 Bergen, Norway; 2 Faculty of Business and Economics, Hawassa University. PO Box 278, Hawassa, Ethiopia; 3 Arbaminch Hospital, Arba Minch, Ethiopia; 4 Department of Public Health and Primary Health Care, University of Bergen, Bergen, Norway

## Abstract

**Background:**

Little is known about the costs of HIV care in Ethiopia.

**Objective:**

To estimate the average per person year (PPY) cost of care for HIV patients with and without anti-retroviral therapy (ART) in a district hospital.

**Methods:**

Data on costs and utilization of HIV-related services were taken from Arba Minch Hospital (AMH) in southern Ethiopia. Mean annual outpatient and inpatient costs and corresponding 95% confidence intervals (CI) were calculated. We adopted a district hospital perspective and focused on hospital costs.

**Findings:**

PPY average (95% CI) costs under ART were US$235.44 (US$218.11–252.78) and US$29.44 (US$24.30–34.58) for outpatient and inpatient care, respectively. Estimates for the non-ART condition were US$38.12 (US$34.36–41.88) and US$80.88 (US$63.66–98.11) for outpatient and inpatient care, respectively. The major cost driver under the ART scheme was cost of ART drugs, whereas it was inpatient care and treatment in the non-ART scheme.

**Conclusion:**

The cost profile of ART at a district hospital level may be useful in the planning and budgeting of implementing ART programs in Ethiopia. Further studies that focus on patient costs are warranted to capture all patterns of service use and relevant costs. Economic evaluations combining cost estimates with clinical outcomes would be useful for ranking of ART services.

## Background

The prevalence of HIV in adults in Ethiopia is 2.1% according to 2007 estimates [1]. About 242,548 adults living with HIV/AIDS require anti-retroviral therapy (ART) [1]. Ethiopia launched a nationwide ART program in January 2005 [2] with a policy to implement treatment to rural settings through peripheral healthcare facilities. The number of treatment sites had reached 272 by June 2007 [1]. Treatment coverage is about 35% [1], and the unmet need for ART in Ethiopia remains considerable.

ART provision in Ethiopia is funded mainly through external programs such as the Global Fund to Fight AIDS, Tuberculosis and Malaria; the United States President's Emergency Plan For AIDS Relief; and other donor agencies. External funding may not continue at its current level as HIV becomes less of an emergency and more of a chronic problem [3]. Ethiopia may therefore have to assume the major share of the cost of care and treatment in the future. This may involve policies and strategies that ensure the cost-effectiveness and sustainability of the intervention. A costing study of ART provision in Ethiopia may offer useful insights into the functioning of such treatment, and provide a premise for policy analysis and debates. Specifically, cost analysis may create a ground for economic evaluations through identification of inputs and their valuations. It also provides an overview of the cost profile of different components of an intervention, and the total amount of resources needed to sustain or expand a project [4].

Several region- and country-specific cost estimates of HIV-related services have been reported, though cost estimates from Africa are meagre. Bertozzi *et al *[5] reported a per person year (PPY) cost of US$538 for ART-based care in low-income countries based on data from sub-Saharan African countries. A study from South Africa [6] stated that the cost of ART decreased compared with a non-ART condition; it also reported an average PPY cost ranging from US$950 to US$3,520 for a non-ART condition, and US$793 to US$964 under ART. Another study from South Africa indicated an average PPY cost of US$580–956 for the non-ART condition, and US$700–2192 under the ART scheme [7]. A recent report from Haiti suggested that ART costs approximately US$1000 PPY, of which 36% goes to anti-retroviral (ARV) drugs [8].

In Ethiopia, cost analysis of HIV-related care and treatment will have direct relevance to the implementation of ART in rural settings because of the dearth of information on ART cost. Our study aimed to estimate the average PPY cost of care of HIV patients with and without ART in a district hospital.

## Method

### Study setting

Our study was done at Arba Minch Hospital (AMH) in the Southern Nation Nationalities and Peoples Region (SNNPR) in Ethiopia. We received from the SNNPR Health Bureau ethical clearance and permission to access documents and patient records.

AMH has 158 beds and serves 1.5 million people. The hospital started HIV-related interventions in the early 1990s [9]. The HIV Unit in AMH was upgraded in January 2002; and it started to offer ART in August 2003. The Ethiopian government launched a nationwide program with free provision of ART in October 2005, and AMH became part of this scheme.

Patient selection and the ART regimen at AMH followed national recommendations and those set by the World Health Organaization (WHO) [10-12]. AMH provided treatment on an outpatient basis (though AIDS patients with severe clinical manifestations could be admitted). First-line drugs for adults included (Stavudine_40 mg or 30 mg_-Lamivudine_150 mg_-Neverapine_200 _mg,), (Zudovudine_300 mg_-Lamivudine_150 mg_-Neverapine_200 mg_), (Stavudine_40 mg or 30 mg_-Lamivudine_150 mg_-Efavirenz_600 mg_) and (Zudovudine_300 mg_-Lamivudine_150 mg_-Efavirenz_600 mg_) [11]. Patients were staged according to clinical manifestation, presence of AIDS-defining illnesses, and basic laboratory tests. Complete blood cell (CBC) count and clinical chemistry have been standard laboratory tests for HIV patients at AMH since January 2003. CD4 count was introduced in September 2005, but there was no viral load analyzer. The HIV Clinic had one physician, one nurse, one data clerk and two community healthworkers. The data clerk maintained the Clinic database; the community healthworkers monitored patients and made regular home visits. AMH maintained a database of HIV patients who received care and treatment.

### Data collection

#### Cost data

Costing was done from the perspective of AMH, and included outpatient and inpatient costs using an ingredient approach. Costs were estimated for direct capital and recurrent inputs for final HIV-related services; shares from overhead cost centres of the hospital (see below) were also included.

Final services in relation to HIV care at AMH included outpatient consultations at the HIV Clinic, laboratory tests, imaging, drug provision, and inpatient services. Costs incurred in providing these services were direct costs. Costs of other work units of the hospital that facilitated provision of final services comprised the overhead costs of final services. Major overhead cost centres were administration, maintenance, storage, medical records, pharmacy, transport, domestic services (i.e., cleaning, security), laundry, clothing, food and utilities. The Ethiopian fiscal year starting 8 July 2004 and ending 7 July 2005 was used as the base year, and cost data were collected retrospectively.

We identified the resource items used in HIV care, including direct and overhead capital and recurrent inputs for the base year. We obtained the 2004/5 price of drugs and medical supplies from the Pharmaceuticals and Medical Supplies Import and Wholesale Share Company (PHARMID) and the SNNPR Health Bureau. We used 2005/6 prices if 2004/5 prices were not available. We retrieved personnel cost and unit costs of each of the non-medical supplies recurrent inputs from the financial records of the accounts section of AMH. For capital inputs, we took the 2005/6 replacement price from the market. We assumed that input costs obtained from the accounts section of AMH, PHARMID, SNNPR Health Bureau, and replacement values of capital items reflected market values of the inputs. All costs were converted to the US dollar using the average exchange rate in 2005 (US$ 1 = ETB 8.6649) [13], and we adjusted the 2005/6 prices to the base year 2004/5 values using the gross domestic product (GDP) deflator for the year 2005/6 [14]. We calculated net present values (NPV) and annuitized the cost of capital items using an interest rate of 3% based on annual yields on government bond [15].

#### Data on service use

We used two prospective cohorts of HIV-positive patients who received care and treatment at AMH from January 2003 to March 2006 to estimate use of HIV-related healthcare services [9,16,17]. The first cohort comprised HIV patients who received care without ART at the HIV Clinic from 1 January 2003 to 8 April 2004 (15.2 months) and a total of 80.81 person year observations (PYO). There were 203 HIV positive patients in this cohort and 181 (89%) were in the non-AIDS state whereas 22 (11%) were in the AIDS state. The second cohort comprised HIV patients who received ART from August 2003 to March 2006 (31.2 months) with 222 PYO. The cohort had 209 HIV positive patients out of which 154 (74%) were in the non-AIDS state whereas 55 (26%) were in the AIDS state. Patients aged <15 years upon enrolment were excluded from the study.

For outpatients, we extracted the following information from the outpatient database of the cohorts: demographic characteristics; date of starting treatment; clinical stage upon starting treatment; investigations done; drugs used; frequency of outpatient visits to the HIV Clinic; and time of discontinuing care at the HIV Clinic. Patients who did not attend within 90 days after their next scheduled visit were considered to be "lost to follow-up"

We retrieved inpatient service records for all 58 HIV patients who had been admitted to AMH during 2004/5 because the HIV Clinic database did not contain information regarding inpatient clinical events and service use. Twenty-five patients were on ART and 33 patients were not. We collected the following data from the patient chart: demographic characteristics; number of inpatient days; clinical stage upon admission; investigations done; and ARV and non-ARV drugs used.

### Data Analysis

#### Unit costs of HIV-related services at AMH

We calculated average unit costs of each of the services by dividing the cost of inputs incurred along each of the services during the base year by the total number of output of the respective services during the base year.

We allocated hospital overhead costs to final HIV-related services using a "stepping-down" approach [18]. We used floor area to allocate utilities, maintenance and domestic service costs. The numbers of staff in each department were used to allocate administration and clothing costs; number of patients or quantity of service were used to allocate central store, medical records and pharmacy/dispensary costs to different activities. We allocated transport costs as a function of the direct cost of each of the final service units.

#### Service use and mean annual cost of care

We approximated the use of outpatient-based HIV-related services by the frequency of visits to the HIV Clinic, laboratory and imaging tests each patient underwent during follow-up, and by the quantity and frequency of prescription of ARV and other drugs. We extracted these data from the outpatient database of the HIV Clinic. Mean annual service use, confidence intervals at 95%, and corresponding costs were calculated as ratios of the frequencies of use of each service to the total PYO.

In estimating inpatient costs, we evaluated annual use of inpatient services for the 58 HIV patients (33 non-ART and 25 ART) who were admitted during the fiscal year 2004/5. Variables associated with inpatient service included the number of non-ARV drugs taken, investigations (laboratory, imaging) done, general inpatient care, and meals. The cost of drugs and investigations were estimated based on the amount consumed by each patient, but we used the number of inpatient days to estimate the cost of general inpatient care and for treatment other than drugs and diagnostics. We then multiplied the quantity of service each patient used with the respective unit cost to obtain the annual cost of inpatient service for each patient. Mean PPY cost and the corresponding 95% confidence interval were estimated from aggregated data.

We used the CostIt [19] spreadsheet to categorize and summarize data of hospital costs, and SPSS 14.1 software for statistical analysis of patient data.

## Results

### Unit costs

Table 1 shows the direct, overhead and aggregate unit costs of HIV-related hospital services for the base year 2004/5. CD4 test was the most expensive HIV-related service (cost, US$6.8 per unit) whereas other laboratory tests (e.g., stool, sputum, blood film) were the cheapest (cost, US$0.9 per unit). The overhead share of unit costs ranged from 6% for CD4 count to 56% for outpatient consultation. The capital component appeared to contribute the major share of the unit cost for CD4 count, haematology test, and imaging examination with 85%, 61% and 56% of the totals, respectively. Recurrent costs were the major components of the outpatient consultation, clinical chemistry and other laboratory tests, and accounted for 78%, 71% and 61% of the totals, respectively.

**Table 1:** Unit cost of HIV-related services at AMH for the base year 2004/5 in US $ ($ 1 = 8.6649 ETB)

	**Direct unit costs**			**Overhead unit costs**			**Combined**		
			
**Cost items**	**Recurrent**	**Capital.**	**Total**	**Recurrent**	**Capital.**	**Total**	**Recurrent**	**Capital**	**Total**
**Consultation at HIV Clinic**	1.13	0.19	1.32	1.18	0.48	1.66	2.31	0.67	**2.98**
**Laboratory**				0.24^1^	0.17^1^	0.41^1^			
Haematology (CBC)	0.50	0.98	1.48	0.24	0.17	0.41	0.74	1.14	**1.88**
Clinical chemistry	0.77	0.48	1.25	0.24	0.17	0.41	1.01	0.64	**1.65**
CD4	0.79	5.72	6.51	0.24	0.17	0.41	1.03	5.89	**6.92**
Other tests	0.39	0.07	0.46	0.24	0.17	0.41	0.62	0.24	**0.87**
**Imaging**	0.64	1.72	2.37	1.33	0.81	2.13	1.97	2.53	**4.50**
**Inpatient services^2^**									
Treatment and care (medical ward)	1.11	0.29	1.40	1.01	0.33	1.34	2.12	0.61	**2.74**
Meal (all wards)				0.77	0.13	0.90	0.77	0.13	**0.90**

^1^Overhead costs were calculated by dividing the total laboratory overhead cost by the total number of laboratory tests during the base year. The recurrent and capital unit overhead costs therefore remained constant for all types of test.

^2^Unit costs of inpatient services are expressed as costs per inpatient days.

### Use of hospital service

Table 2 summarizes the mean annual service utilization of HIV patients at AMH. HIV patients who were on ART tended to have more outpatient consultations and clinical chemistry tests than those who were not on ART. Mean annual use of imaging, and outpatient-based haematology tests appeared to be higher in the non-ART group than in the ART group. Use of inpatient-based laboratory tests appeared to be similar in the two groups. Patients on ART had fewer inpatient days than those who were not on ART.

**Table 2:** Average annual per patient use (95% CI) of hospital services by ART and AIDS status at AMH (2004/5)

	**Non-ART**			**ART**		
	
**Services categories**	**Total**	**non-AIDS**	**AIDS**	**Total**	**non-AIDS**	**AIDS**
**Outpatient services**	PYO* = 80.82	PYO = 75.24	PYO = 5.58	PYO = 221.98	PYO = 181.13	PYO = 40.85
***Consultation at HIV Clinic***	**4.6(4.3–5.0)**	**4.5(4.1–4.9)**	**5.9(4.0–7.9)**	**11.4(11.0–11.7)**	**11.4(11.0–11.8)**	**11.3(10.5–12.1)**
***Laboratory***						
*Haematology (CBC)*	4.4(4.0–4.8)	4.3(3.9–4.9)	5.4(3.5–7.3)	3.1(2.96–3.2)	3(2.9–3.2)	3.3(2.9–3.7)
*Clinical chemistry*	1.1(0.9–1.3)	1.1(0.8–1.3)	1.3(0.1–2.4)	3.1(2.9–3.2)	3.1(2.9–3.2)	3.2(2.8–3.6)
*CD4*	0.0	0	0	0.4(0.3–0.4)	0.4(0.3–0.4)	0.3(0.2–0.5)
*Other tests*	0.3(0.2–0.5)	0.3(0.2–0.4)	0.7(0.1–1.6)	0.3(0.2–0.4)	0.3(0.2–0.4)	0.4(0.2–0.5)
***Imaging***	0.7(0.5–0.9)	0.6(0.5–0.8)	1.6(0.6–2.6)	0.1(0.05–0.13)	0.1(0.0–0.1)	0.1(0.0–0.2)
***Anti Retroviral drugs***						
*Zudovudine (300 mg)*	0.0	0	0	28.7(13.3–44.1)	33.4(14.6–52.1)	8.3(0.0–16.7)
*3TC (150 mg)*	0.0	0	0	548.2(515.2–581.1)	551.1(515.2–587.0)	535.0(451–618.9)
*Stavudine (d4T; 40 mg)*	0.0	0	0	205.1(162.4–247.8)	224.3(175.3–273.2)	120.4(44.9–195.8)
*Stavudine (d4T; 30 mg)*	0.0	0	0	317.7(268.2–367.1)	297.7(242.6–352.8)	406.2(294.7–517.8)
*Neverapine (200 mg)*	0.0	0	0	401.2(353–449.4)	402.8(349.4–456.1)	394.3(277.9–510.7)
*Effavirenze (600 mg)*	0.0	0	0	113.3(89.6–137.0)	112.1(85.7–138.4)	118.9(63–174.8)
*Zudovidine+3TC 450 mg*	0.0	0	0	88.3(59.9–116.7)	88.6(57.2–120.1)	86.8(18.8–154.8)
*Non-ARV drugs*^1^	10.9(8.4–13.3)	10.0(7.6–12.4)	22.4(7.8–37.0)	3.8(3.1–4.5)	3.9(3.0–4.7)	3.5(2.6–4.5)

**Inpatient services**	n = 33	n = 13	n = 20	n = 25	n = 19	n = 6
***Laboratory***						
*Haematology (CBC)*	0.9(0.7–1.0)	0.9(0.5–1.2)	0.9(0.6–1.1)	0.7(0.5–0.9)	0.7(0.5–1.0)	0.7(0.1–1.2)
*Clinical chemistry*	0.4(0.2–0.6)	0.3(0.1–0.7)	0.5(0.2–0.7)	0.4(0.2–0.7)	0.5(0.2–0.7)	0.3(-0.2–0.9)
*CD4*	0.00	0	0	0.08(0.03–0.19)	0.1(0.05–0.3)	0
*Other tests*	1.3(0.8–1.7)	1.31(0.5–2.1)	1.3(0.7–1.6)	0.5(0.1–0.9)	0.5(0.1–0.9)	0.7(0.6–1.9)
***Imaging***	0.5(0.3–0.7)	0.4(0.01–0.8)	0.6(0.3–0.9)	0.08(0.03–0.19)	0.1(0.06–0.16)	0.17(0.06–0.60)
***Non-ARVdrug*^1^**	11.3(8.8–13.9)	11.2(7.7–14.7)	11.4(7.6–15.2)	5.5(3.9–7.1)	5.7(3.7–7.6)	5.0(0.9–9.0)
***Mean number of inpatient days***	17.6(13.1–22.1)	12.2(6.2–18.1)	21.1(14.9–27.3)	5.6(4.4–6.9	4.5(3.5–5.6)	9.2(5.7–12.6)

*Person year observation

^1 ^Utilization is expressed in terms of US $.

Except in the use of outpatient-based non-ARV drugs in the absence of ART and the number of inpatient days under non-ART and ART conditions, it appeared that there was no major difference in service use between the non-AIDS and AIDS states within each treatment condition. AIDS patients not on ART used more non-ARV drugs than those patients without AIDS, whereas this was comparable for those who were on ART. Under both treatment conditions, those in the latter stage of AIDS had more inpatient days than those without AIDS.

### Mean annual cost of care and treatment of HIV

Table 3 shows the average PPY cost of outpatient and inpatient HIV care at AMH. Overall costs of inpatient care appeared to be higher under the non-ART condition than the ART condition, but the cost of outpatient-based services was higher under the ART situation. The PPY costs of care without ART were US$38 and US$81 for outpatient and inpatient services, respectively. With ART, outpatient costs increased to US$235, whereas inpatient costs decreased to US$29. Cost of ARV drugs was the major cost driver under ART (78% of outpatient costs). There are large differences in costs between patients depending on whether or not they have AIDS for all inpatients services and for all outpatients not receiving ART. Except for the costs of outpatient services to those receiving ART, both the outpatient and inpatient costs are higher for patients with AIDS within both the non-ART and ART conditions.

**Table 3:** Average per patient year cost (95% CI) of HIV related services in US$* at Arba Minch Hospital by ART and AIDS status (2004/5)

	**Non-ART**			**ART**		
		
**Cost categories**	**Total**	**Non-AIDS**	**AIDS**	**Total**	**Non-AIDS**	**AIDS**
	
**Outpatient services**						
***HIV Clinic consultation***	13.8(12.7–14.9)	13.5(12.4–14.6)	17.6(11.8–23.4)	33.9(32.9–35.0)	33.9(32.8–35.1)	33.7(31.4–36.0)
***Laboratory***	10.3(9.3–11.3)	10.1(9.1–11.2)	12.8(7.5–18.2)	13.7(12.9–14.4)	13.5(12.7–14.3)	14.4(12.5–16.2)
***Imaging***	3.2(2.4–3.9)	2.9(2.2–3.6)	7.3(2.8–11.7)	0.4(0.2–0.6)	0.4(0.2–0.6)	0.3(0.04–0.7)
***ARV drugs***	0.00	0	0	183.7(167.0–200.4)	183.9(165.6–202.3)	182.7(140.9–224.5)
***Non-ARV drugs***	10.9(8.4–13.3)	10.0(7.6–12.4)	22.4(7.8–37.0)	3.8(3.1–4.5)	3.9(3.0–4.7)	3.5(2.6–4.5)
						
**Total per patient annual cost of outpatient care**	38.1(34.4–41.9)	36.5(32.8–40.2)	60.1(35.5–84.6)	235.4(218.1–252.8)	235.6(216.5–254.8)	234.6(192.5–276.7)
						
**Inpatient services**						
***Laboratory***	3.4(2.7–4.0)	3.2(2.1–4.4)	3.4(2.5–4.3)	3.1(2.1–4.1)	3.3(2.1–4.5)	2.4(0.4–4.4)
***Imaging***	2.3(1.3–3.3)	1.7(0.04–3.5)	2.7(1.4–4.0)	0.4(0.2–0.9)	0.24(0.2–0.7	0.8(0.18–2.68)
***Non-ARV drugs***	11.3(8.8–13.9)	11.2(7.7–14.7)	11.4(7.6–15.2)	5.5(3.9–7.1)	5.7(3.7–7.6)	5.0(0.9–9.0)
***Treatment and care***	48.1(35.9–60.3)	33.2(17.1–49.4)	57.7(40.8–74.6)	15.4(11.9–19.0)	12.4(9.6–15.2)	25.1(15.6–34.6)
***Meals***	15.8(11.8–19.9)	10.9(5,6–16.3)	19.0(13.4–24.6)	5.1(3.9–6.2)	4.1(3.2–5.0)	8.3(5.1–11.4)
						
**Total per patient annual cost of inpatient care**	80.9(63.7–98.1)	60.4(35.2–85.5)	94.2(71.3–117.2)	29.4(24.3–34.6)	25.7(20.9–30.4)	41.4(27.8–55.1)

## Discussion

### Principal finding

We found that the cost of care under ART appeared to be higher than that under the non-ART condition in a district hospital setting in Ethiopia. Mean annual outpatient and inpatient costs of treating HIV patients with ART were US$265, and US$119 without ART. The cost of ARV drugs was the major cost element, and accounted for >70% of the annual cost under ART. Inpatient care and treatment was the most important cost if patients did not receive ART.

### Discussion of main findings

Unit costs derived in our study (i.e., US$2.98 per outpatient visits and US$3.64 per inpatient day) were higher than those reported by WHO for secondary-level hospitals in Ethiopia for year 2000. WHO values were US$0.43 per outpatient visit and US$1.77 per inpatient day [20]. Our estimates were service-specific, whereas WHO estimates were aggregates for all services, which may explain the difference. Nevertheless, unit costs in our study were less than those reported from a recent study in South Africa. Cleary *et al*. [7] reported unit costs of US$18.92 and US$19.33 for an outpatient clinic visit under non-ART and ART conditions, respectively. This is probably because South Africa is a medium-income country with a higher level of general cost than Ethiopia.

Mean estimates of PPY outpatient visits in our study for non-AIDS and AIDS patients (4.5 and 5.9, respectively, and 4.6 combined) under the non-ART condition were similar to those found in other studies. A study from Mexico reported pre-ART mean annual outpatient visits of 4.6–6.3 [21]. A study from South-Africa [6] estimated an average PPY outpatient visit of 4.35 and 6.6 for non-AIDS and AIDS groups, respectively, under the non-ART condition. Our estimates of outpatient visits for non-AIDS and AIDS categories under ART (11.37 and 11.31, respectively, and 11.36 combined) were less than the 15 visits PPY reported in a study from Haiti [8] (though slightly higher than the Mexican and South African studies). The study from Mexico reported post-ART PPY outpatient visits of 8.9–10.3 [21], whereas the South African study reported PPY outpatient visits of 8.71 and 7.62 for the non-AIDS and AIDS categories, respectively, under the ART scenario [6].

Our estimates of PPY inpatient days appeared higher under non-ART and ART conditions than the estimates from the South African and Mexican studies. The South African study reported PPY inpatient days of 3.75 and 15.36 for non-AIDS and AIDS stages, respectively, under the non-ART condition; and 1.08 and 2.04 for non-AIDS and AIDS states, respectively, under the ART scenario. Estimates from the Mexican study were even lower: PPY inpatient days were 0.7–2.2 in the pre-ART period, and 1.3–1.9 in the post-ART period.

Total costs of HIV care and treatment in our study were more favourable than earlier studies [6-8], with annual per patient costs of outpatient and inpatient services being significantly lower. Direct comparison of cost values from different settings may not be straightforward due to different assumptions and study designs, but cost values in our study may have appeared favourable because Ethiopia is a poor country with low levels of income, and relatively low prices of domestic inputs.

### Study limitations

We applied an ingredient approach for cost estimation, so most of the inputs for final HIV-related services and overhead activities were considered in the cost estimation. Nevertheless, certain limitations and shortcomings in our costing approach are evident.

First, we applied average (unit) cost in estimating service costs, which is the commonest approach in costing studies of health services. Such estimation of unit cost is likely to be affected by the quantity of service delivered during a specified period [18]. Service categories that operate outside their optimum capacity are therefore likely to have higher unit costs. The HIV Clinic at AMH probably treated fewer patients than its capacity because ART was introduced in Ethiopia recently and coverage is low. Estimated unit costs may therefore have been overstated. The alternative could be estimating each of the inputs required to provide each service to a single patient.

Second, we applied identical unit costs of service for non-ART and ART conditions. The intensity of use of services under the two scenarios could differ, and consequently the unit cost of delivering the services may vary, as indicated by Cleary *et al *[7].

Third, our estimation of in-patient costs was based on a small sample of patients and data on service use for a single year. This resulted in fewer patients in the AIDS and non-aids categories; and the limited duration of follow-up may not capture the pattern of service use and the corresponding cost over several years. This might affect the precision of the estimates and the result may need to be interpreted carefully. Although small samples may affect precision of estimates, we calculated confidence intervals, and believe our results represent important information for utilization of the data in economic evaluation models.

Our limited sample size was because of the difficulty of retrieving retrospective in-patient service-use data for HIV patients at AMH. We had to retrieve data on in-patient service use of HIV patients from the general patient records (which we could get for only a single year) because the HIV database at AMH focused on outpatient care and treatment follow-up. Thus, it may be important for healthcare facilities to keep a comprehensive database that covers all clinical events of patients under their care.

## Conclusion

In spite of its limitations, our study highlighted the average cost profile of ART in a district hospital in Ethiopia, and the results may have direct application for program planning. At a district hospital, on average, about US$235 and US$30 PPY must be spent for outpatient and inpatient care, respectively, for patients on ART. This is more than twice as high as the costs of non-ART services. This finding indicates that an economic evaluation of ART, in Ethiopia, would be valuable to consider if incremental costs per incremental life years is reasonable value-for-money.

Our cost estimates are important information for the implementation of ART in Ethiopia, but further studies that focus on patient costs may be warranted to capture all patterns of service use.

## Competing interests

The authors declare that they have no competing interests.

## Authors' contributions

ADB designed the study, analyzed the data, and wrote the manuscript. DJ established the AMH cohort database and helped to edit the manuscript. BR contributed to study design, data analysis, writing and approving the manuscript. BL contributed to the conception and design of the study; data analysis, writing and approving the manuscript
